# Habitat‐dependent occupancy and movement in a migrant songbird highlights the importance of mangroves and forested lagoons in Panama and Colombia

**DOI:** 10.1002/ece3.5610

**Published:** 2019-09-26

**Authors:** Lesley Bulluck, Elizabeth Ames, Nicholas Bayly, Jessie Reese, Cathy Viverette, James Wright, Angela Caguazango, Christopher Tonra

**Affiliations:** ^1^ Center for Environmental Studies Virginia Commonwealth University Richmond VA USA; ^2^ School of the Environment and Natural Resources The Ohio State University Columbus OH USA; ^3^ SELVA: Investigación para la Conservación en el Neotropico Bogotá D.C. Colombia; ^4^ Department of Biology Virginia Commonwealth University Richmond VA USA

**Keywords:** abundance, cienaga, dynamic occupancy, mangrove, overwintering, persistence, prothonotary warbler

## Abstract

Climate change is predicted to impact tropical mangrove forests due to decreased rainfall, sea‐level rise, and increased seasonality of flooding. Such changes are likely to influence habitat quality for migratory songbirds occupying mangrove wetlands during the tropical dry season. Overwintering habitat quality is known to be associated with fitness in migratory songbirds, yet studies have focused primarily on territorial species. Little is known about the ecology of nonterritorial species that may display more complex movement patterns within and among habitats of differing quality. In this study, we assess within‐season survival and movement at two spatio‐temporal scales of a nonterritorial overwintering bird, the prothonotary warbler (*Protonotaria citrea*), that depends on mangroves and tropical lowland forests. Specifically, we (a) estimated within‐patch survival and persistence over a six‐week period using radio‐tagged birds in central Panama and (b) modeled abundance and occupancy dynamics at survey points throughout eastern Panama and northern Colombia as the dry season progressed. We found that site persistence was highest in mangroves; however, the probability of survival did not differ among habitats. The probability of warbler occupancy increased with canopy cover, and wet habitats were least likely to experience local extinction as the dry season progressed. We also found that warbler abundance is highest in forests with the tallest canopies. This study is one of the first to demonstrate habitat‐dependent occupancy and movement in a *nonterritorial* overwintering migrant songbird, and our findings highlight the need to conserve intact, mature mangrove, and lowland forests.

## Graphical abstract

We found that overwintering occupancy and movement are habitat‐dependent in a nonterritorial overwintering migrant bird. Warblers in mangroves and other wet habitats are least likely to experience local extinction as dry season progresses, and warbler abundance was highest in mature mangroves forests. Conserving mangroves and forested wetlands is key to retaining quality winter habitat for migratory birds.

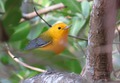

## 1 INTRODUCTION

Many species of migratory birds occupy mangrove and lowland riparian tropical forests during the overwintering period, as they tend to have higher prey abundance than drier habitats (e.g., Chan, Yu, Zhang, & Dudgeon, 2008; Smith, Reitsma, & Marra, 2011; Wunderle, Lebow, White, Currie, & Ewert, 2014). Conservation of migratory species is challenging because their shifting distributions make it difficult to identify the potentially diverse factors limiting populations at different points throughout the annual cycle (Marra, Cohen, Loss, Rutter, & Tonra, 2015; Runge, Martin, Possingham, Willis, & Fuller, 2014; Webster, Marra, Haig, Bensch, & Holmes, 2002). This is especially problematic for the overwintering period, which often encompasses the largest portion of the annual cycle and is generally when the most challenging environmental conditions are experienced (e.g., tropical dry season; Rushing, Ryder, & Marra, 2016; Smith, Reitsma, & Marra, 2010). Moisture is a factor known to influence habitat quality during the overwintering period, and this is especially true for species that rely on lowland habitats such as mangroves, lagoons, and flooded riparian forests. Studies of the territorial Northern Waterthrush in Caribbean mangroves demonstrated that moisture plays a role in mass gain and spring departure date to the breeding grounds (Smith et al., 2010). Likewise, studies with territorial American Redstarts show that moisture‐driven differences in habitat quality influence survival (Johnson, Sherry, Holmes, & Marra, 2006) and can carry over to influence reproductive success in the breeding season (e.g., Reudink et al., 2009). These studies highlight the importance of habitat moisture which varies widely between tropical wet and dry seasons, with the driest times often corresponding to the premigratory period for Neotropical migratory songbirds. Inter‐annual changes in rainfall can also have a significant impact on food, mass change, and spring departure (Studds & Marra, 2007). Understanding how seasonal drying influences changes in habitat‐specific survival, abundance and site persistence will aid in our predictions of how birds may respond to longer term drying trends caused by climate change (Neelin, Munnich, Su, Meyerson, & Holloway, 2006) and will also help to prioritize conservation efforts in declining mangrove and lowland forests.

Much of our current understanding of overwintering ecology in Nearctic‐Neotropical migratory songbirds has been based on studies of habitat‐specific demography in *territorial* populations (e.g., American Redstart *Setophaga ruticilla*, Marra, 2000; Ovenbird *Seiurus aurocapilla*, Brown & Sherry, 2006). Nonterritorial species (i.e., those that flock, maintain a nonexclusive home range, or are transient) represent an additional overwintering strategy that needs further study as they display more complex movement patterns than individuals maintaining an exclusive territory. As a result, effective metrics of habitat quality are likely to differ between territorial and nonterritorial species. For example, differences in age/sex ratios between habitats can be the result of despotic distributions of highly territorial species (Fretwell & Lucas, 1970), and not likely relevant for nonterritorial species. Density (Johnson, 2007) and site persistence (Latta, Howell, Dettling, & Cormier, 2012) are likely to be indicators of habitat quality for nonterritorial species. Density can be a cue for resource availability (Stamps, 1991) and nonterritorial birds are more likely to move out of (i.e., not persist in) habitats that decline in quality over time compared to species that have invested time in establishing a territory. Within‐season movements are likely more common than previously recognized; studies in Panama (Lefebvre, Poulin, & McNeil, 1992), Belize (Gómez & Bayly, 2010), and at multiple sites from Venezuela to Mexico (Ruiz‐Gutierrez, Kendall, Saraco, & White, 2016) suggest some species move between habitats/regions as the tropical dry season progresses. Recent evidence of large‐scale intra‐tropical migration has also been observed in some species that are thought to avoid competition or track resources to increase their chances for survival (Koleček et al., 2018; Stutchbury et al., 2016). Despite our understanding of the occurrence of within‐season movements of overwintering birds, few studies have examined habitat‐related factors that may be driving these intra‐seasonal movements (but see Smith et al., 2011; Wunderle et al., 2014).

In this study, we assess within‐season survival, site persistence, and movement of the prothonotary warbler (*Protonotaria citrea*) in central Panama and quantify abundance and occupancy dynamics across a broader region of the known wintering range in Panama and northern Colombia. Our goal was to assess whether demography and site persistence varied as a function of habitat in this nonterritorial species (Lefebvre, Poulin, & McNeil, 1994; Morton, 1980; Warkentin & Hernandez, 1996). In Panama, we deployed radio transmitters in habitats that varied in the level of disturbance and moisture to assess movement patterns of individuals at small temporal and spatial scales. We also modeled abundance and occupancy dynamics of prothonotary warblers across Panama and Colombia between early, wetter months (November to December) and later, drier months (January to February) to assess broadscale shifts in occupancy among habitats. We predict that at both the local and regional spatial scales, wetter mangrove and lowland forest habitats will be of better quality to overwintering birds. Specifically, we expect survival, abundance, and occupancy to be higher in wetter compared with drier habitats and that birds will be more likely to exhibit site persistence as the dry season progresses in wetter habitats.

## 2 MATERIALS AND METHODS

### 2.1 Focal species

**Figure 1:**
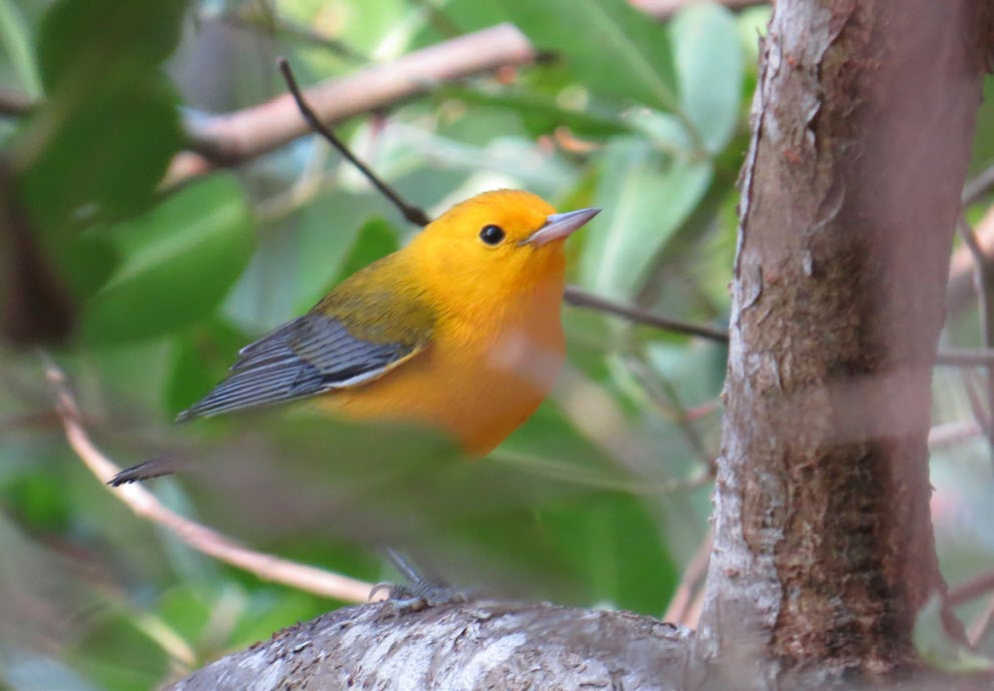
Male prothonotary warbler in Salamanca National Park, Colombia. Photograph taken by Nick Bayly

The prothonotary warbler (Figure 1) is a Neotropical migrant songbird that breeds throughout eastern North America and overwinters in Central and northern South America. The prothonotary warbler population has declined by about 1% per year over large portions of the breeding range since the 1960s (Sauer et al., 2015) with declines reaching 5.5% per year in some years (Ziolkowski, Pardieck, & Sauer, 2010). Contemporary declines have occurred despite much of the preferred bottomland forest breeding habitat being cleared prior to 1966 (Dickson, Thompson, Conner, & Franzreb, 1995). However, habitat destruction of mangroves and wet lowland forests on the wintering grounds may be at least partially responsible for contemporary population declines. Because of threats due to habitat loss, continuing population declines, and relatively low population size for such a widespread species, the prothonotary warbler is considered a Bird of Conservation Concern in the United States (USFWS, 2008).

Analysis of light‐level geolocator data from prothonotary warblers breeding in Virginia, South Carolina, Arkansas, Ohio, and Louisiana indicate that most individuals from across these disparate breeding populations overwinter in north‐central Colombia, including the Magdalena River Valley (Tonra et al., 2019). This previously unknown area of importance is further inland than the coastal mangrove forests thought to be the primary overwintering habitat for prothonotary warblers. Few studies employing geolocators have demonstrated such widely separated breeding populations converging on the same overwintering area (*but see* Renfrew et al., 2013; Fraser et al., 2012). A smaller number of individuals also overwintered in Panama where mangroves are being rapidly drained, filled, and developed (Lopez‐Angarita, Roberts, Tilley, Hawkins, & Coole, 2016). These geolocator data have provided a useful starting point but lack spatial precision; on the ground studies assessing the relative quality of habitats are needed to help prioritize and justify conservation efforts in specific areas.

### 2.2 Study area

**Figure 2:**
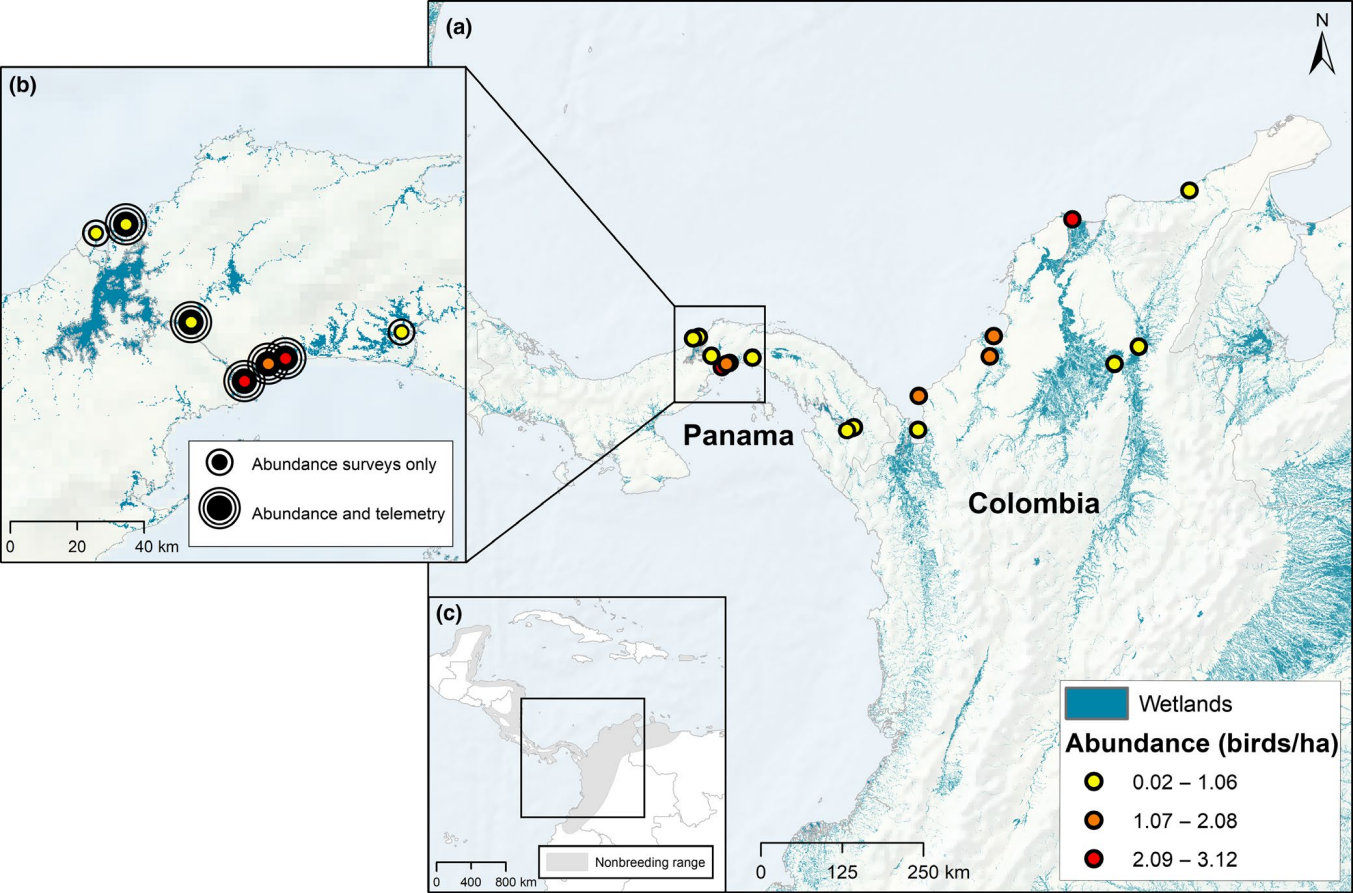
(a) Location of study sites in Panama and Colombia. Point count surveys were conducted at all study sites, and colored points represent estimated mean prothonotary warbler abundance (birds/ha) from the most supported abundance model. See Table 1 for names of sites. (b) Radio telemetry and banding of individuals took place at five sites in Panama. (c) Study occurred within the overwintering range of the prothonotary warbler. Wetlands data shown here for context is courtesy of the Center for International Forestry Research (Gumbricht et al., 2017) and was not used in the analysis

**Figure 3:**
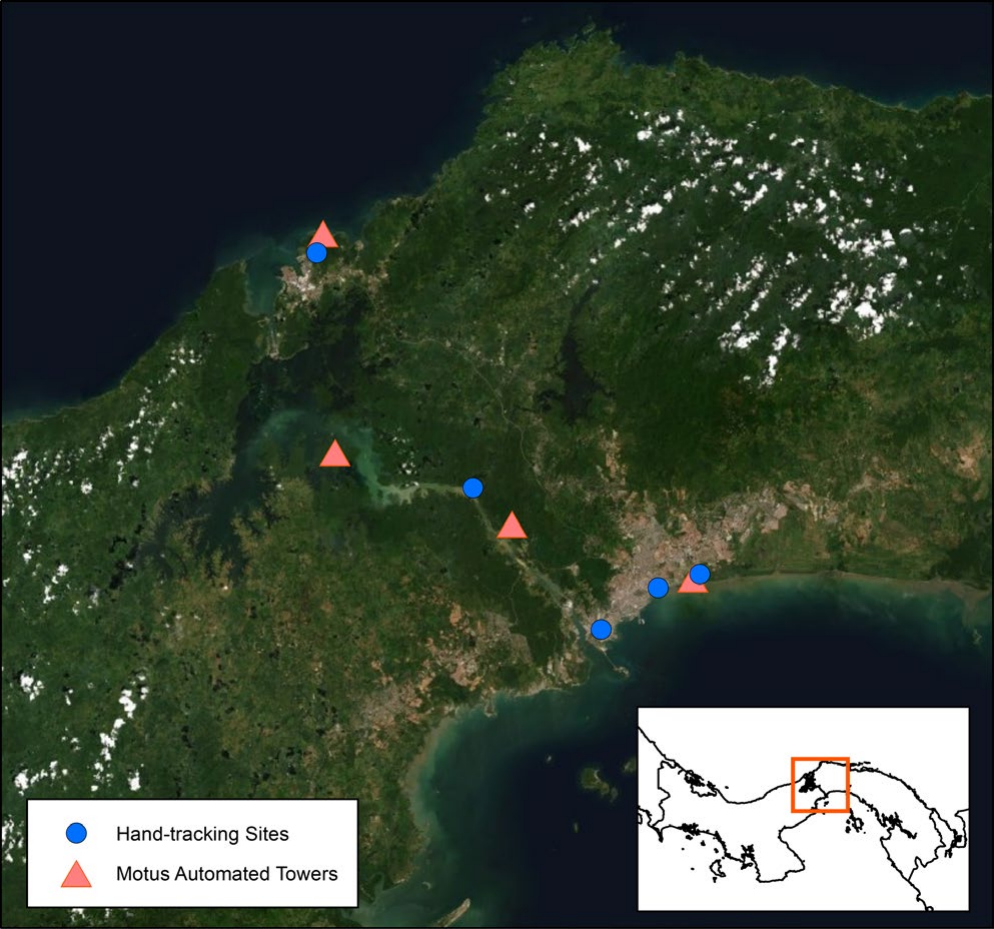
Location of sites in the Panama Canal Region where we tracked individual prothonotary warblers using VHF tags as well as the location of Motus towers that could detect larger scale movements of these same tagged birds

This study took place across 17 sites, eight in Colombia and nine in Panama. We conducted surveys in all sites and mark–recapture and telemetry of individual birds at a subset of five sites in Panama (Table 1, Figures 2 and 3). Ten sites were primarily comprised of habitat typically associated with prothonotary warblers during the overwintering period—mangrove forests and lagoons (cienagas). Mangrove sites were often a mix of black and white mangrove (*Avicennia germinans* and *Laguncularia racemosa*, respectively) with other less common mangrove species occasionally present (*Avicennia bicolor*). Some mangrove sites (i.e., Galeta on the Caribbean coast of Panama) had a higher proportion of red mangrove (*Rhizophora mangle*). The remaining sites were a combination of habitats where prothonotary warblers also occur—forested freshwater wetlands associated with rivers (i.e., Rio Bayana and Rio Pirre in Panama and Rio Magdalena in Colombia) and secondary forests disturbed by clearing for agriculture or development adjacent to inland rivers or mangrove sites. All sites represented a gradient of habitat moisture ranging from wet mangrove and cienaga forests that tended to stay wet throughout the overwintering period to freshwater wetlands and secondary forests that tended to dry out as the dry season progressed.

**Table 1:** List of study sites in Panama and Colombia arranged from west to east, number of survey locations, whether it was a telemetry site, and the primary and secondary habitat type

Site	Country	# points (# surveyed twice)	Telemetry?	Primary habitat	Secondary habitat
Ciénaga Bañó	Colombia	20 (19)	N	Cienaga	
Bocas del Atrato	Colombia	19 (19)	N	Mangrove	
Cispatá	Colombia	10 (10)	N	Mangrove	
Flamencos	Colombia	18 (18)	N	Mangrove	
Reserva El Garcero	Colombia	18 (18)	N	FW wetlands	Cienaga
Ciénaga de Marimonda	Colombia	18 (18)	N	Cienaga	
Salamanca	Colombia	18 (18)	N	Mangrove	
Ciénaga de Zapatosa	Colombia	16 (16)	N	Cienaga	
Rio Bayano	Panama	37 (8)	N	FW wetlands	Mangrove
Cerro Ancon	Panama	5 (0)	Y	Secondary forests	
Galeta Research Station	Panama	29 (20)	Y	Mangrove	FW wetlands
Gamboa	Panama	4 (0)	Y	Secondary forests	
Juan Diaz	Panama	9 (5)	Y	Mangrove	Secondary forests
Panama Viejo	Panama	6 (2)	Y	Mangrove	Secondary forests
Rio Pirre	Panama	26 (7)	N	Secondary forests	FW wetlands
San Lorenzo	Panama	31 (7)	N	FW wetlands	Mangrove
Rio Tuira	Panama	29(9)	N	Secondary forests	

Note

The number of points indicates the sample used for abundance models and the number of sites surveyed twice indicates the sample used for occupancy models. For sites used for radio telemetry, whether they were categorized as wet or dry sites is indicated (see Section 2 for further description), as well as the number of birds tracked in parenthesis.

### 2.3 Abundance and occupancy surveys

Point count surveys were carried out across all sites in Panama and Colombia during the overwintering season (November 2016 to February 2017, Figure 2). We conducted surveys after the prolonged fall migration and prior to spring migration (Tonra et al., 2019). Each site had 4 to 37‐point locations, and many points (62%) were surveyed twice, once at the beginning of the dry season (November/early December) and again in the middle of the dry season (late January/early February). We placed the majority of survey points in mangroves and forests surrounding ciѐnagas, and the remainder in forested freshwater wetlands and secondary forests (Table 1). We classified points as either mangrove, cienaga, freshwater wetland, or secondary forest and used this habitat classifier as a covariate in occupancy and abundance models (see analysis section below).

Survey points were 50 m fixed radius and at least 250 m apart, often much farther, to avoid multiple detections of individuals. Before the survey began, we used a laser rangefinder (Nikon 550) to determine the distance of landmarks to aid in visualizing the edges of the 50 m radius survey area. All surveys were conducted between sunrise and 10 a.m. Each survey lasted eight minutes and was divided into four 2‐min survey periods where individual birds could be recorded more than once if detected in more than one period. This method allowed for estimation of detection probability from one survey using the capture–recapture framework (see Section 2.5 of survey data below). Either directly after the survey or later that day, surveyors recorded basic habitat information at each survey point. Specifically, we recorded forest type and type of water present, visually estimated percent canopy cover and measured canopy height of the five closest canopy trees (taller than 2 m) using a laser rangefinder (Nikon 550). The canopy height covariate used in models is the average of these five trees.

### 2.4 Individual movement, site persistence, and survival

We assessed movement, survival, and site persistence at five study sites in the Panama Canal Region (Figure 3) from 18th December 2016 to 7th February 2017. We used mist nets to passively and actively (distress calls and chips broadcasted) capture prothonotary warblers. We banded all birds with a USGS aluminum band and a unique color band combination for field identification. For each individual captured, we recorded mass (±0.01 g), structural body size (i.e., wing, tail, and tarsus length), age (young = within first year of life, or adult = after first year), and sex. To control mass for structural body size and time of capture (i.e., potential time of day sampling bias), we created a body mass index (hereafter BMI) using the fitted values from a linear model with wing chord, time of capture, and a wing chord*time of capture interaction term (*N* = 84, adjusted *R* ^2^ = .473). This model produced the lowest Akaike's Information Criterion (AIC) value when compared to other models that included wing chord, time of capture, age, and sex (closest model was ΔAIC = 3.92).

To track warbler movements and survival, we deployed digital nanotag radio transmitters (Lotek Wireless model NTBQ‐2, Inc.; tag warranty life 54 days) on individuals across the five sites. We used hand tracking (homing and triangulation) to determine overwinter survival and site persistence by visiting each site at least once every 5 days and locating tagged individuals during each visit. We exhaustively searched surrounding habitat to locate individuals when not found in the main study area. We considered a disappearance from the study area a movement, and a recovered tag with signs of predation or death to be a mortality event. In addition to hand tracking, each nanotag is individually identifiable within one VHF frequency, allowing them to be detected by Motus automated telemetry towers (Motus Wildlife Tracking System, Bird Studies Canada, http://motus-wts.org/; Taylor et al., 2017). We collected Motus data from four automated stations in the Panama Canal Zone to identify landscape level movements. For prothonotary warblers foraging in dense mangrove habitat, we estimated the detection range of the Motus stations to be reliable up to approximately 400 m by using known locations of radio‐tagged birds (i.e., hand tracking data) to compare detections. Beyond 400 m birds would likely only be detected in open habitat or exiting the forest. We filtered raw Motus data by removing detections with run lengths <2 (run lengths of 2 were only considered with other supporting detections), pulse lengths that differed from our tags (9 s), unlikely locations (i.e., towers outside the winter range), and ambiguous tags. Data were then visually inspected to ensure detections were highly plausible.

All research activities in Panama were approved by the USGS Bird Banding Lab (Permit 23941), a Scientific Research Permit from the Panama Ministry of Environment (MiAmbiente; Permit SE/A‐123‐16), and the Institutional Animal Care and Use Committees of the Smithsonian Tropical Research Institute (Protocol 2016‐1215‐2019) and the Ohio State University (Protocol 201500000028). Research in Colombia did not involve capture of birds and observation protocols were approved under Resolución No. 179‐2015 from Parques Nacionales Naturales de Colombia and Resolución 0597‐2014 and 0189‐2016 from Autoridad Nacional de Licencias Ambientales (ANLA) issued to SELVA.

### 2.5 Statistical analysis

#### 2.5.1 Abundance and occupancy estimation

Point‐specific abundance was estimated using a capture–recapture model with the multinomPois function (Chandler, 2015) of Package Unmarked (Fiske & Chandler, 2011) in program R (v 3.4.1; R Core Team, 2017). This function can simultaneously model variation in detection probability and abundance following the framework established by Royle (2004) and Dorazio, Jelks, and Jordan (2005). All data were stacked for the abundance analysis meaning that each season (early dry and late dry) is considered a “new” point and you can therefore model time variables as site covariates. This allows for explicit testing of a season effect and prevents having to run the models twice in order to assess whether there are significant changes in abundance over time.

Dynamic occupancy models estimate the probability of occupancy, colonization, and local extinction as a function of covariates (MacKenzie et al., 2003) using the colext function in Package Unmarked (Fiske & Chandler, 2011) in program R. Only point locations that were surveyed more than once were used in this analysis (*N* = 176). Secondary forests were excluded from occupancy models because of the small number of points surveyed more than once (*N* = 18) and the limited range of canopy cover recorded in this habitat, which impacted our ability to model the influence of a habitat*canopy cover interaction. Secondary forests were the driest habitats, and we might expect them to experience the highest rates of local extinction as they undergo significant transformation in the dry season with some species of trees losing their leaves. Our study includes tracking of individual birds in these habitats which should inform this expectation in the absence of dynamic occupancy results.

Before modeling abundance or occupancy, we modeled the detection process to see if there were any predictors that explained variation in our ability to detect prothonotary warblers. We specifically looked at time of day, day of the year, canopy height, and canopy cover as potential predictors of detection. Detection probability is related to a bird's activity level which can decrease with increasing temperature and as a function of seasonal changes in behavior (Buckland, Anderson, Burnham, & Laake, 1993). We included canopy height and cover as potential detection covariates because vegetation structure can also influence detectability of birds (Pacifici, Simons, & Pollock, 2008). We expected prothonotary warbler detection to decrease with increasing canopy height and decreasing canopy cover because the birds would be farther away from the observer and in denser vegetation, respectively. Few studies have assessed such changes in detection for overwintering birds. Detection probability was modeled separately for abundance and occupancy models because the datasets were different for these analyses (Table 1). Any factor(s) that influenced detection were carried over to abundance and occupancy models. The following univariate covariates were used as potential predictors to explain variation in abundance and occupancy: habitat type, canopy cover, canopy height, and date. We used canopy cover as a proxy for disturbance and canopy height as a proxy for forest age and expected that abundance would be positively related to forest age and negatively related to the degree of disturbance. Because we thought the influence of forest age and disturbance may not be consistent across habitats, we also compared models with an interaction between habitat and canopy height and an interaction between habitat and canopy cover. These six models were compared using AIC. For abundance models, a model with country as a covariate (Panama/Colombia) was also used.

Any factors that influenced detection and occupancy were carried over to models of colonization and extinction. Colonization and extinction were not modeled simultaneously; no covariates were included for extinction in the colonization models, and no covariates were included for colonization in extinction models.

#### 2.5.2 Individual movement, site persistence, and survival

We used radio transmitter data to estimate site persistence and within‐season survival. To do this, we combined sites by moisture level (i.e., wet = standing water was observed throughout the study; dry = either no standing water or the site dried up within three weeks) and habitat type (i.e., mangrove or nonmangrove, Table 1) and used these as predictors for statistical analyses to explore differences in survival and site persistence among habitats. Site persistence and survival were estimated using Program MARK known fate models (White & Burnham, 1999). To estimate differences in both site persistence and survival rates between the two habitat types (mangrove vs. nonmangrove) and sites with persistent rather than ephemeral moisture (wet vs. dry), we created two models, one for habitat type and one for moisture level using a grouping variable. To estimate individual predictors, we used likelihood ratio tests to test the significance of each predictor by removing it from the global model (i.e., grouping variable, time since tagging, age, sex, and BMI) and comparing the reduced model to the global model. Noninformative covariates in preliminary analyses (likelihood ratio test all *p* > .071) were removed from the final model. The inclusion of time since tagging represents a fully time‐dependent model, where site persistence/survival could vary between each tracking period. We included BMI as it has been shown to indicate resource availability and impact overwinter survival (Wolfe, Johnson, & Ralph, 2013). We expected birds in mangrove and wet habitat to have higher BMI and that this would positively impact survival and persistence. To explore potential differences in survival and persistence between age and sex groups, we included these variables in the models as well. For survival estimates, nonsite persistent birds were right‐censored after departure. For site persistence estimates, we right‐censored confirmed mortalities (*n* = 3), recovered tags with signs of predation or death, and considered a disappearance from the study area a departure from the site.

To generate locations from triangulation data, we used LOAS software (Ecological Software Solutions LLC) and excluded locations with error >35 m. For each radio‐tagged bird, we calculated a maximum distance moved (between any two of their locations) to gauge the span of distance moved during the study period, and we calculated an average of those across all individuals at a site. We also calculated straight line travel distance for each consecutive hand tracking location (Matthews & Rodewald, 2010) for each bird with >5 locations. Due to data collection restraints, we were not able to obtain enough points to perform home range analysis. For consecutive distances, only locations generated from tracking sessions >1 day apart were used, most were 3–5 days apart, and they were not significantly correlated to length of time between tracking sessions (Pearson's correlation = 0.048, *p*‐value = .52). As the raw distribution for consecutive distances was heavily right skewed, we log‐transformed it to achieve a normal distribution and analyzed it using generalized linear mixed effects models in a Bayesian framework with the “brms” package in Program R (v 3.4.1; R Core Team, 2017). We constructed each model to predict distance between consecutive locations with a random effect for individual bird and fixed effects for: habitat type or moisture level, age, sex, and BMI. Model inference was based on 15,000 Markov chain Monte Carlo draws from four parallel chains, with uninformative priors (burn‐in = 5,000; thin = 4). We used leave‐one‐out (LOO) cross‐validation and widely applicable information criterion (WAIC) to compare a priori models and select a best fitting model. We estimated Bayesian R squared (Bayes_*R* ^2^; Gelman, Goodrich, Gabry, & Ali, 2017) values for each model within the “brms” package.

## 3 RESULTS

### 3.1 Overwintering abundance in Colombia and Panama

In Colombia, 150 total points were surveyed (137 in the early dry season, 136 in the middle of the dry season) and 136 (90.7%) were surveyed in both seasons. In Panama, 168 total points were surveyed (109 in the early dry season, 117 in the middle of the dry season) and 58 (34.5%) were surveyed in both seasons. Fewer points were surveyed twice in Panama because the first round of surveys took place along roads, and a subset were moved away from the road for the second round.

The top detection model included canopy cover and had an AIC weight of 0.41; the null model with no factors was the second‐best model (ΔAIC = 1.41), suggesting that canopy cover is not a strong influence on detection (95% CI = −0.0003, 0.008). Detection probability was generally low and ranged from 0.40 when canopy cover is <25% to 0.47 when canopy cover is >75%. Despite the weak effect of canopy cover on prothonotary warbler detection, we accounted for it by including it in all models of abundance.

**Figure 4:**
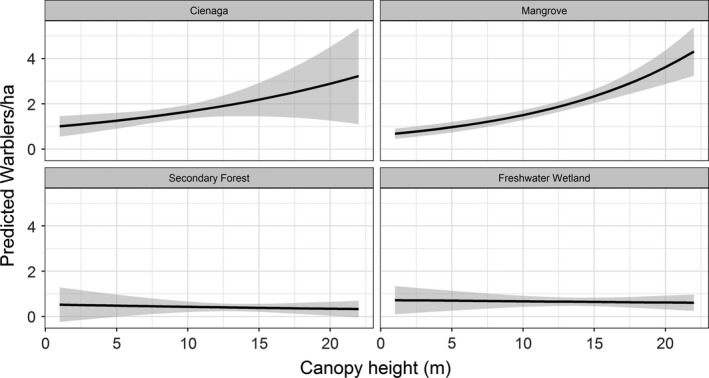
Prothonotary warbler abundance is correlated with canopy height in mangroves and lagoons (cienagas) with higher abundance of prothonotary warbler present in forests with taller canopies. This same relationship does not exist in freshwater wetland and secondary growth forests where abundance is generally lower. Shaded regions represent 95% confidence intervals

The most supported model for prothonotary warbler abundance included an interaction between habitat type and canopy height (Table 2) and had an AIC weight of 1.00. Specifically, canopy height produced a fourfold increase in prothonotary warbler abundance but only in mangrove and cienaga habitats (5 m canopy = 1 ind/ha; 20 m canopy = 4 ind/ha); both secondary forest and freshwater wetlands had low and uniform abundances regardless of canopy height (1 ind/ha, Figure 4).

**Table 2:** AIC comparison for models of Prothonotary Warbler abundance (Lambda)

Abundance models	nPars	AIC	Delta	Cum AICwt
Habitat*canopy height	10	3,430.32	0.00	1.00
Habitat*canopy cover	10	3,445.45	15.13	1.00
Habitat	6	3,476.54	46.220	1.00
Country	4	3,521.24	90.92	1.00
Canopy cover	4	3,543.11	112.79	1.00
Canopy height	4	3,573.14	142.83	1.00
Null (no predictors)	3	3,581.68	151.37	1.00
Date	4	3,586.36	156.04	1.00

Note

All models include a canopy cover covariate for detection probability. nPars is the number of parameters in the model, AIC is the Akaike Information Criterion value, delta is the difference in AIC values between that model and the top performing model, and Cum AICwt is the cumulative AIC weight.

### 3.2 Dynamic occupancy in Colombia and Panama

Prothonotary warbler detection probability in the occupancy dataset is positively correlated with forest canopy height (*p* = .0009, 95% CI = 0.026, 0.102). While we originally hypothesized warbler detection to be negatively influenced by canopy height, detection probability was lowest in forests with canopies between 5 and 10 m (*p* = ~.55) and higher in forests with canopies between 15 and 20 m (*p* = ~.75). This could be due to taller stature habitats being more open, so that observing/hearing individual birds is more likely compared with shorter, and denser, canopy structures. The second most supported model was the null model (ΔAIC = 9.58), suggesting that no other factors that we measured adequately describe variation in warbler detection. In the dataset used to model warbler abundance, there was a relationship between detection probability and canopy cover, not canopy height. The most plausible explanation is that the abundance and occupancy datasets are very different; *N* = ~300 points in abundance models and *N* = ~180 points for occupancy models (those that were surveyed twice, the majority of which were in Colombia).

**Figure 5:**
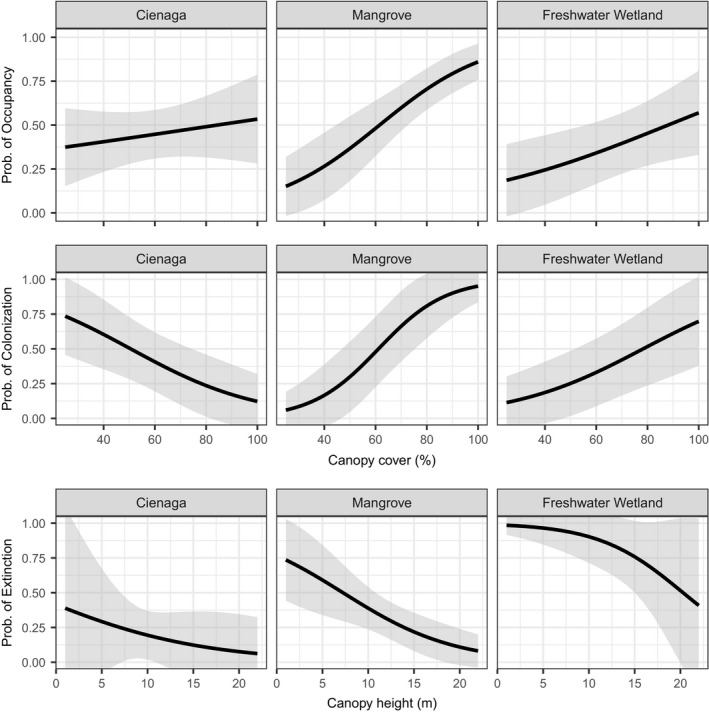
Predicted probability of prothonotary warbler occupancy, colonization, and extinction from the top performing models of these processes. Occupancy and the probability of prothonotary warbler colonization between the wet and dry season are best explained by an interaction between habitat type and percent canopy cover. The probability of prothonotary warbler extinction is best explained by an interaction between habitat and canopy height. Shaded regions represent 95% confidence intervals

The top model describing prothonotary warbler occupancy included an interaction between habitat type and canopy cover (Table 3). The second‐best model (ΔAIC = 2.07) includes only canopy cover. Across all three habitat types (secondary forests were excluded, see Section 2), the probability of warbler occupancy increased with increasing canopy cover and this relationship was strongest in mangroves, followed by freshwater wetlands and cienagas (Figure 5).

**Table 3:** AIC comparison for models of Prothonotary Warbler occupancy

Occupancy models	nPars	AIC	Delta	Cum AICwt
Habitat*Canopy cover	10	1,406.49	0.00	0.71
Canopy cover	6	1,408.56	2.07	0.96
Habitat*Canopy height	10	1,412.49	6.00	1.00
Canopy height	6	1,416.80	10.31	1.00
Habitat	7	1,423.80	17.31	1.00
Null model	5	1,429.14	22.65	1.00

Note

All models include a canopy height covariate for detection probability. nPars is the number of parameters in the model, AIC is the Akaike Information Criterion value, delta is the difference in AIC values between that model and the top performing model, and Cum AICwt is the cumulative AIC weight.

The best model describing colonization as the dry season progressed also included an interaction between habitat type and canopy cover and performs much better than all other models (Table 4, ΔAIC > 13). The probability of warbler colonization increased with increasing canopy cover in mangroves and freshwater wetlands while the probability of colonization decreased with canopy cover in cienagas (Figure 5). The best model describing extinction probability as the dry season progressed included an interaction between habitat type and canopy height (Table 5). The second‐best model (ΔAIC = 2.79) included habitat type only. Mangroves, cienagas, and freshwater wetlands have higher extinction probabilities when they have low canopy heights. Among these three habitats, mangroves have the lowest extinction probability and freshwater wetlands have the highest (Figure 5).

**Table 4:** Colonization models (accounting for Habitat*canopy cover influence on occupancy and canopy height influence on detection)

Colonization models	nPars	AIC	Delta	Cum AICwt
Habitat*Canopy cover	15	1,390.91	0.00	1.00
Canopy cover	11	1,404.62	13.71	1.00
Null model	10	1,406.49	15.58	1.00
Canopy height	11	1,408.47	17.56	1.00
Habitat	12	1,409.43	18.52	1.00
Habitat*Canopy height	15	1,413.99	23.09	1.00

**Table 5:** Extinction models (accounting for canopy cover influence on occupancy as well as the canopy height influence on detection)

Extinction models	nPars	AIC	Delta	Cum AICwt
Habitat*Canopy height	15	1,394.73	0.00	0.73
Habitat	12	1,397.52	2.79	0.91
Habitat*Canopy cover	15	1,399.03	4.30	0.99
Canopy height	11	1,404.78	10.05	1.00
Null model	10	1,406.49	11.76	1.00
Canopy cover	11	1,408.35	13.62	1.00

### 3.3 Winter site persistence and survival in Panama

A total of 87 prothonotary warblers were captured during the study and 29 individuals received nanotags. Over the duration of the study, we confirmed mortality for three nanotagged warblers: one in reptile scat with feathers, one mangled on the ground with plucked feathers (probable avian predator), and one mangled in a tree (unknown predator but possibly avian). Survival was generally high across all habitats and the best model predicting survival consisted solely of time since tagging (Table 6). The best model for predicting site persistence in mangrove versus nonmangrove habitat contained time since tagging, BMI, and sex. The inclusion of time since tagging in the final model suggests that site persistence varied between tracking intervals. BMI was positively correlated with site persistence and females were more likely to persist than males. The estimated probability that an individual remained in mangrove habitat was 20.9% higher than in nonmangrove habitat. When comparing wet versus dry habitat, the best model for site persistence contained time since tagging and sex (again females were more likely to persist than males), and site persistence was 13.2% greater in wet than dry habitat (Table 6).

**Table 6:** Prothonotary warbler site persistence estimates and survival estimates, from late December 2016 to early February 2017, using the best fitting model for mangrove versus nonmangrove habitat and wet versus dry habitat, with lower 95% confidence interval (LCI 95%) and upper 95% confidence interval (UCI 95%)

Parameter	Site persistence estimate	LCI 95%	UCI 95%	Survival Estimate	LCI 95%	UCI 95%
Mangrove habitat	0.831	0.559	0.951	0.827	0.508	0.956
Nonmangrove habitat	0.622	0.292	0.868	0.903	0.541	0.987
Wet habitat	0.809	0.468	0.953	0.884	0.487	0.984
Dry habitat	0.677	0.402	0.868	0.848	0.553	0.964

### 3.4 Winter movement in Panama

#### 3.4.1 Landscape level movement

We detected one landscape level movement with the Motus tower array. This tag was deployed on a young bird in a small secondary forest patch adjacent to the Rio Chagres (Gamboa site) on December 30th and was subsequently detected through 9th January. After 9th January, the bird was not detected again until it was picked up by a Motus automated array 29 km south in a large mangrove forest adjacent to the Rio Juan Diaz on 2nd, 9th, and 28th February. Multiple detections of this individual in the same area suggest that it remained in the area for an extended period of time. No other large‐scale movements (>1.5 km) were detected by the Motus towers.

### 3.5 Site level movement

The maximum distance moved for each individual, over the duration of the study (i.e., 6 weeks), ranged from 36 m to 1,223 m. The average maximum distance moved was lowest at the two wet mangrove sites (mean = 141.44, *SE* = 23.16 and mean = 174.65, *SE* = 34.42) with the dry secondary site having an intermediate value (mean = 239.73, *SE* = 36.88), and highest at the dry mangrove site (mean = 418.44, *SE* = 102.87) and the secondary forest site (mean = 425.85, *SE* = 175.33). Distances between consecutive tracking locations ranged from 0 m (i.e., individual in same location as previous observation period) to 1,149 m. Consecutive distances were lowest at the two wet mangrove sites (mean = 47.21, *SE* = 5.03 and mean = 71.22, *SE* = 6.49) with the dry secondary site having similar values (mean = 73.87, *SE* = 8.25), and highest at the dry mangrove site (mean = 96.45, *SE* = 22.06) and the secondary forest site (mean = 193.42, *SE* = 52.90).

**Figure 6:**
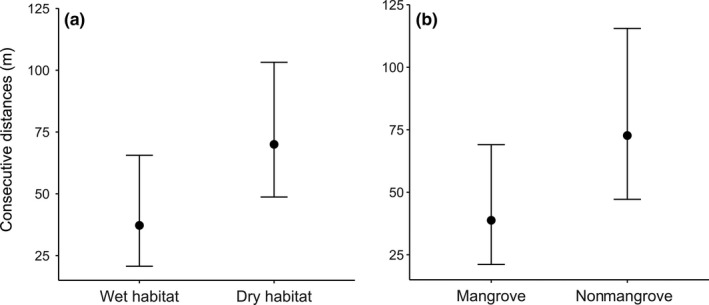
Predicted consecutive distances (meters) between locations for prothonotary warblers, from late December 2016 to early February 2017, for the two best Bayesian linear mixed effects models from leave‐one‐out cross‐validation. (a) The wet habitat model and (b) The mangrove habitat model. Error bars represent 95% credible interval

The best model for consecutive distance contained moisture level as the only predictor. It explained 23% of the variation with 91.8% confidence that birds from wet habitat were found closer to their previous location than birds in dry habitat (Bayes_*R* ^2^ = .23, overlap with 0 = 8.25%, βwet = −0.38, 95% credible interval [−0.95, 0.17]). On average, birds in wet habitat moved 37 m (95% credible interval [20.7, 65.6]) and birds in dry habitat moved 70 m (95% credible interval [48.7, 103.2]) between tracking periods (Figure 6). The second‐best model, which contained only habitat type as a predictor, also explained 23% of the variation for consecutive distance traveled with 88.8% confidence that birds from mangrove habitat were closer to their previous location than birds in nonmangrove habitat (Bayes_*R* ^2^ = .23, overlap with 0 = 11.2%, βmangrove = −0.34, 95% credible interval [−0.93, 0.22]; Figure 6).

## 4 DISCUSSION

Multiple habitat quality indicators independently support the importance of wet, mature forests for overwintering prothonotary warblers. This study enhances our understanding of overwintering habitat use and movement of a nonterritorial songbird by combining data collected at two spatio‐temporal scales. Both broadscale surveys and local tracking of birds indicate that wetter habitats, specifically mature, undisturbed mangroves (i.e., with more canopy cover), are higher quality habitats for prothonotary warblers than drier, disturbed habitats. These findings suggest that overwintering habitat quality varies significantly and mediates within‐season movements. Our study is one of the first to demonstrate that winter site persistence and occupancy dynamics, recently found to be more variable than once thought (Gumbricht et al., 2017), can be correlated with habitat in a nonterritorial migrant songbird.

The importance of moisture to mangrove‐associated species has been well established in overwintering territorial birds (Johnson et al., 2006; Marra, Hobson, & Holmes, 1998; McKinnon, Rotenberg, & Stutchbury, 2015; Norris et al., 2004; Smith et al., 2010; Studds & Marra, 2005). This suggests that wet habitats buffer individuals against the effects of seasonal drought which is predicted to become more intense with climate change (Neelin et al., 2006). Smith et al. (2011) found that as the Caribbean dry season progressed, birds in habitats prone to drying were more likely to make home range shifts to wetter areas compared with birds in habitats less prone to drying, possibly representing a form of resource tracking. As prothonotary warblers are also wet forest specialists, we expected to see higher site fidelity and lower probability of extinction in wetter habitats. Despite our small sample size of radio‐tracked birds, the best performing site persistence model supports this idea—estimated site persistence in mangrove habitat was 20.9% higher than in nonmangrove habitat. The inclusion of time since tagging in the top model indicates that site persistence varied over the course of the study, which may be indicative of varying moisture levels and drying rates in the different habitats. Further supporting this idea, we found that the probability of warbler occupancy was highest in wetter habitats (mangroves and cienagas) and the probability of extinction as the dry season progressed was lowest in these same habitats. Birds with higher BMI when initially captured, before significant drying had occurred, had higher rates of site persistence suggesting either that their home ranges encompassed more available resources, or alternatively they were buffered against a reduction in resources (i.e., seasonal drying trends) and thus able to persist at the site longer than other birds. More information is needed to determine if the higher persistence rate of females is indicative of differing physiological requirements of the sexes, social dynamics, or other sex‐specific constraints.

The importance of moisture was further supported by data on movement distances of individually tracked birds across different habitat types. We found that small scale movements are likely related to habitat moisture, as birds in nonmangrove and dry sites had both greater maximum and consecutive location distances than individuals in mangrove and wet sites. There were no sex or age differences in consecutive distance traveled, suggesting that habitat impacts are consistent regardless of potential dominance hierarchies (e.g., Marra, 2000). We were only able to explain 23% of the variance in our system suggesting that there are additional, unmeasured factors that influence movement patterns. We also documented two landscape level relocations (>1 km), which represented (a) a movement from nonmangrove to mangrove habitat (29 km) and (b) a movement from dry nonmangrove forest to wet nonmangrove forest (1.2 km). In addition, it is likely that many of the birds that were not site persistent moved greater than 1 km from the study site as the vicinity adjacent to the study area was thoroughly searched when birds were not located during the tracking survey.

Mature wet forests (mangroves and forests surrounding cienagas with 15–20 m tall canopies) with the least amount of disturbance appear to be most important for overwintering prothonotary warblers as they had higher probability of occupancy and supported higher abundances compared to secondary forests and freshwater wetlands. While density is not always an indicator of habitat quality (Van Horne, 1983), it can be more likely to indicate resource availability for nonterritorial and flocking species such as the prothonotary warbler. Further supporting the quality of mature forests, the probability of extinction as the dry season progressed was driven by canopy height, where shorter stature (i.e., younger) forests were more likely to experience local extinction than taller stature forests. Consistent occupation of sites throughout the overwintering period indicates that important resources are likely to be present which are not found in sites that become unoccupied. This is especially important during the premigratory period (late February/early March) when birds need to acquire fat reserves to fuel northward migration. Our survey results demonstrate that cienaga and mangrove habitats remain important throughout the prothonotary warbler overwintering period. Cienagas with less canopy cover were more likely to be colonized between the wet and dry seasons, and the opposite was true for mangroves and freshwater wetlands—those with more canopy cover were likely to be colonized. This may be because cienaga habitats with open canopies are indicative of more standing water, whereas standing water is present under closed canopy in mangrove and freshwater wetland habitats. Overall, while some research has emphasized the value of disturbed habitats to overwintering migrants (e.g., shade coffee farms; Johnson & Sherry, 2001), our findings add to a body of literature demonstrating that in many species mature forests are of greater conservation value, based on bird numbers (e.g., Bayly, Rosenberg, Gomez, & Hobson, 2019; Cespedes & Bayly, 2018) and the amount of resources available (e.g., Smith & Robertson, 2008).

A recent study by Ruiz‐Gutierrez et al. (2016) using banding data from a network of stations throughout Mexico, Central and South America showed that it was common for prothonotary warblers to be transient rather than resident between November and March. Interestingly, the trend for winter persistence varied with latitude where southerly latitudes (Panama and Colombia) were more likely to have site persistence (i.e., not experience local extinction) than higher latitude sites (i.e., Belize to Nicaragua; Ruiz‐Gutierrez et al., 2016). It is possible that a prolonged southerly fall migration period, which has been documented with geolocator data (Tonra et al., 2019), could explain transient birds in more northerly latitudes. There is a need for more studies to link site persistence with habitat, as we have done here, because it provides an additional metric for ranking sites for conservation (Ruiz‐Gutierrez et al., 2016).

## 5 CONCLUSIONS

Understanding prothonotary warbler movement patterns and habitat use during the overwintering period and identifying abundance hotspots is important for prioritizing conservation efforts for the species. The high abundance of prothonotary warblers at Colombian sites compared to many Panamanian sites indicates it is an important overwintering area for a significant portion of the global population. The importance of this region in supporting individuals from across the species' breeding range is supported by migratory connectivity research using stable isotopes (Reese et al., in review), population genetics (DeSaix et al., 2019), and geolocators (Tonra et al., 2019). Prioritizing habitat conservation in this region may benefit the largest proportion of the global population; however, it is also important to continue to identify additional areas of high use during the overwintering season as there may be areas that have yet to be identified. Studies (Calvert, Woodcock, & McCracken, 2010; Lefebvre & Poulin, 1996; Wolfe et al., 2013) and citizen science data (Sullivan et al., 2009) suggest substantial population densities of prothonotary warblers in other countries (Panama, Costa Rica, and Nicaragua), prompting the need for more surveys in varying habitats across the known wintering range. eBird data are helpful and will inevitably play a role in our understanding of species distributions. However, mangroves and other flooded forests, due to their inaccessibility to most birdwatchers, will continue to be underrepresented and present a bias in our understanding of the true distribution of this and other mangrove‐dependent species. Within high‐abundance areas in Colombia and Panama, our research demonstrates the importance of conserving high quality, mature mangrove forests, and other wet habitats surrounding cienagas, as abundance and persistence was greatest in those habitats. Salamanca National Park in Colombia has the highest mean abundance of prothonotary warblers at 3–4 birds/ha; with 12,000 ha of mangrove cover, this one park is likely to support 36,000–48,000 overwintering birds, or 2%–3% of the global population.

As the Neotropical dry season progressed, mangrove habitat retained more birds and those birds moved less than those in nonmangrove habitat. This is likely because mangroves and cienagas retain more moisture while soils and vegetation in secondary forests and freshwater wetlands dry out, leaving fewer resources (i.e., phytophagous and aquatic emergent arthropods) for birds occupying those habitats (Smith et al., 2011). Focusing conservation efforts on high quality, wet mangroves would provide habitat for the greatest number of birds; however, conserving secondary forests and freshwater wetlands, especially those adjacent to mangroves, would also provide useful habitat. Mangrove forests are facing ever increasing threats from deforestation for development, aquaculture, rising sea levels and reduced precipitation from climate change (Neelin et al., 2006), and anthropogenic changes to hydrologic regimes (Sandilyan & Kathiresan, 2012). Therefore, it is imperative that conservation action be taken to preserve remaining mangroves across the Americas as they provide important overwintering habitat for prothonotary warblers and myriad other terrestrial and aquatic species (Nagelkerken et al., 2008).

The results presented here, coupled with recent publications documenting intra‐tropical migration (Koleček et al., 2018; Stutchbury et al., 2016) and decreased residence times (Ruiz‐Gutierrez et al., 2016), highlight that the ecology of overwintering migratory birds is not as simple (or stationary) as once thought. These findings are leading to a paradigm shift in how we think about the overwintering portion of the annual cycle that has been largely influenced by a focus on stationary and territorial species. The use of site persistence and residence times as measures of habitat quality is not restricted to migratory birds; indeed, studies from butterflies (Shahabuddin, Herzner, & Aponte, 2000) to chimpanzees (Foerster et al., 2016) have found similar relationships. Full life cycle models will need to incorporate the transient nature of species if they are to effectively identify priority areas for conservation (Stutchbury et al., 2016), and further research is required to determine the benefits/disadvantages of occupying more than one habitat. Indeed, prothonotary warblers occupying dry forests on arrival to the overwintering grounds may be taking advantage of seasonal abundances related to the wet season across northern Colombia and Panama (September–November).

## CONFLICT OF INTEREST

None declared.

## AUTHOR CONTRIBUTIONS

Lesley Bulluck, Elizabeth Ames, Christopher Tonra, Nicholas Bayly, Jessie Reese, and James Wright contributed to this manuscript during its conception, analysis, writing, revisions, and final approval. Lesley Bulluck and Elizabeth Ames wrote the first draft of the publication with input from Jessie Reese. Angela Caguazango, Jessie Reese, and James Wright also conducted data collection. Cathy Viverette contributed to this manuscript during its conception, revision, and final approval.

## Data Availability

Data available from the Dryad Digital Repository: https://doi.org/10.5061/dryad.8m63m78
